# Perceived discrimination during the childbirth hospitalization and postpartum visit attendance and content: Evidence from the Listening to Mothers in California survey

**DOI:** 10.1371/journal.pone.0253055

**Published:** 2021-06-23

**Authors:** Laura B. Attanasio, Brittany L. Ranchoff, Kimberley H. Geissler

**Affiliations:** University of Massachusetts Amherst School of Public Health and Health Sciences, Amherst, Massachusetts, United States of America; University of Mississippi Medical Center, UNITED STATES

## Abstract

**Objective:**

Postpartum visits are an important opportunity to address ongoing maternal health. Experiences of discrimination in healthcare can impact healthcare use, including postpartum visits. However, it is unknown whether discrimination is associated with postpartum visit content. This study aimed to examine the relationship between perceived discrimination during the childbirth hospitalization and postpartum visit attendance and content.

**Research design:**

Data were from Listening to Mothers in California, a population-based survey of people with a singleton hospital birth in California in 2016. Adjusted logistic regression models estimated the association between perceived discrimination during the childbirth hospitalization and 1) postpartum visit attendance, and 2) topics addressed at the postpartum visit (birth control, depression and breastfeeding) for those who attended.

**Results:**

90.6% of women attended a postpartum visit, and 8.6% reported discrimination during the childbirth hospitalization. In adjusted models, any discrimination and insurance-based discrimination were associated with 7 and 10 percentage point (pp) lower predicted probabilities of attending a postpartum visit, respectively. There was a 7pp lower predicted probability of discussing birth control for women who had experienced discrimination (81% vs. 88%), a 15pp lower predicted probability of being asked about depression (64% vs. 79%), and a 9 pp lower predicted probability of being asked about breastfeeding (57% vs. 66%).

**Conclusions:**

Amid heightened attention to the importance of postpartum care, there is a need to better understand determinants of postpartum care quality. Our findings highlight the potential consequences of healthcare discrimination in the perinatal period, including lower quality of postpartum care.

## Introduction

For the nearly four million people that give birth in the United States each year [1], attending a comprehensive postpartum visit provides an important opportunity to address health issues resulting from pregnancy and birth, develop a management plan for any chronic conditions, and foster health promotion [2]. Although a 2018 statement from the American College of Obstetricians and Gynecologists (ACOG) reconceptualizes postpartum care as a continuum, a “comprehensive” postpartum visit is recommended by 12 weeks postpartum [3].

Estimates of postpartum visit attendance range from 50% to nearly 90% [4–7]. Previous research has shown that women of color, uninsured women, younger women, women with lower socioeconomic status, and women with delayed prenatal care are less likely to attend postpartum visits [4,7–9]. Previous research has shown that reasons for not attending a postpartum visit include not feeling that more care is needed, being busy with other things including caring for a newborn, as well as access barriers such as not having insurance or transportation to the appointment [10–13]. Experiences of discrimination within the healthcare system are associated with disengagement from healthcare and avoiding seeking needed care, and discrimination may be more common among women from marginalized social groups [14–18]. Indeed, women who experienced discrimination during the childbirth hospitalization were less likely to attend a postpartum visit in a national survey of women who gave birth in 2011–2012 [19]. However, this association has not been confirmed in a population-based sample or in more recent years.

Furthermore, healthcare utilization does not necessarily translate into the receipt of high-quality care that adheres to current professional guidelines. In prenatal care, for example, attending the recommended number of prenatal visits is insufficient for achieving good perinatal outcomes [20–23]; this finding has led to increasing interest in assessing the content and quality of prenatal care [24–26]. A recent study found that most recommended services were delivered at less than half of comprehensive postpartum visits, although there were few differences in provision of recommended services by whether or not the woman was insured by Medicaid [27]. Experiences of discrimination in healthcare generally or during pregnancy and childbirth specifically could affect the quality of care received in the postpartum visit by negatively impacting patient-clinician communication and adherence to recommendations and follow-up [28–31]. However, no studies have explicitly examined whether postpartum visit content varies by experiences of discrimination.

The primary goal of this study was to examine the relationship between perceived discrimination during the childbirth hospitalization and postpartum visit content in a recent population-based sample of women who gave birth in California. We also sought to confirm earlier findings that women who experienced discrimination during the childbirth hospitalization had lower postpartum visit attendance, and to determine reasons for not attending postpartum visits.

## Methods

### Data

We used data from the Listening to Mothers in California survey, a stratified random sample of women who had a singleton hospital birth in California between Sept. 1, 2016 and Dec. 15, 2016. Eligible women were aged 18 years and older, residents of California, and able to complete the survey in English or Spanish. Surveys could be completed online, by phone, or a combination. Black women, women with midwife-attended births, and women with a vaginal birth after cesarean were oversampled. The final response rate was 54%, which is similar to response rates for other population-based surveys [32,33]. Sampling weights were constructed to adjust for non-response and to make the sample representative of births to eligible women in California. Full details of the survey methodology are available elsewhere [34]. Data from this survey have previously been used to examine women’s experiences in intrapartum care [35–38]. This study is not considered human subjects research, as it uses publicly available, de-identified data.

### Measures

We constructed a measure of postpartum visit attendance based on a question about how many office visits the woman had with a maternity care provider between hospital discharge and 8 weeks after the birth. Women who had one or more office visits were categorized as having a postpartum visit.

Postpartum visit content for those who attended a postpartum visit was assessed based on responses to the following three questions: (1) “During your postpartum office [visit/visits] in the first 8 weeks after birth, did any maternity care provider ask if you needed help with a method of birth control?”, (2) “During your postpartum office [visit/visits] in the first 8 weeks after birth, did any maternity care provider ask if you were feeling depressed?”, and (3) “During your postpartum office [visit/visits] in the first 8 weeks after birth, did any maternity care provider ask if you needed help with breastfeeding?” The third question was limited to women who reported that they were breastfeeding at 1 week postpartum.

Women who did not attend a postpartum visit were asked to identify the main reason that they did not attend a visit, with five options: (1) “I didn’t need more care,” (2) “I didn’t have insurance for the visit,” (3) “I didn’t have a way to get to the visit,” (4) “I didn’t feel well/was tired/didn’t want to go out,” or (5) “I had other things to do and didn’t have time.” We created a binary variable for “didn’t need more care” vs. all other reasons.

Discrimination is typically conceptualized as unfair or differential treatment based on group membership [39,40]. The Listening to Mothers in California survey asked the following three questions about perceived discrimination, with response options of never, sometimes, usually, or always: (1) “During your recent hospital stay when you had your baby, how often were you treated unfairly because of the language you spoke?”, (2) “During your recent hospital stay when you had your baby, how often were you treated unfairly because of the language you spoke?”, and (3) “During your recent hospital stay when you had your baby, how often were you treated unfairly because of the type of health insurance you had or because you didn’t have health insurance?” We created a binary variable for each of these statements (never vs. sometimes/usually/always). Additionally, we created an overall binary indicator of whether the woman reported ever experiencing any of the three types of discrimination.

We constructed the following socio-demographic variables: race/ethnicity (White, Black, Asian/Pacific Islander, Latina, other), education level (less than high school, high school diploma or GED, some college, Bachelor’s degree or higher), insurance type at birth (Medi-Cal, private, other), age category (18–24, 25–39, 30–34, 35+), main language spoken at home (English, Spanish, English and Spanish equally, other). We also constructed variables for pregnancy and birth characteristics: birth mode (vaginal birth, planned cesarean birth, unplanned cesarean birth), and parity (first baby or not), prenatal care provider type (midwife or not), and pre-pregnancy obesity. We excluded observations with missing data for any of the independent or dependent variables. The exception to this was whether or not a woman had pre-pregnancy obesity. Nearly 10% of the sample had a missing value for body mass index; we created an indicator variable for missing obesity status in order to be able to include these observations in the analysis.

### Analysis

We examined differences in postpartum visit attendance by socio-demographic and pregnancy and birth characteristics, using chi-square tests to identify statistically significant differences across groups. We assessed the relationship between perceived discrimination and attending a postpartum visit, estimating multivariable logistic regression models controlling for sociodemographic, pregnancy, and birth characteristics. We then calculated predicted probabilities. Limiting the sample to women who attended a postpartum office visit, we estimated multivariable logistic regression models with binary indicators of each topic potentially addressed at the postpartum visit as outcomes and perceived discrimination as the key predictor, controlling for sociodemographic, pregnancy, and birth characteristics. Using these multivariate models, we estimated predicted probabilities of the outcomes by perceived discrimination. Finally, we limited the sample to women who had not attended a postpartum visit and conducted bivariate analyses examining reasons for not attending a visit by socio-demographic characteristics, pregnancy and birth characteristics, and perceived discrimination. We did not estimate multivariate models with the outcome of reason for not attending a postpartum visit due to the small sample size. All analyses use survey weights to make the results representative of births in California. An alpha of 0.05 was considered statistically significant. All analyses were conducted in Stata 16.

## Results

Overall, 90.6% of women in the sample attended a postpartum visit (Table 1). Postpartum visit attendance was more common among women with private insurance vs. Medi-Cal (5.8% vs. 12.9%, p<0.001), and among women with higher levels of education (p = 0.002). Younger women had lower rates of postpartum visit attendance (p = 0.042). Among women with planned cesarean births, 94.3% attended a postpartum visit, compared to 92.2% of women with unplanned cesarean births and 89.4% of women with vaginal births (p = 0.01).

**Table 1:** Characteristics of women in the Listening to Mothers California survey overall and by postpartum visit attendance (N = 2,295).

		Attended postpartum visit		
	Total	No	Yes	*P*
Total		9.4	90.6	--
Race/ethnicity				0.081
White	27.3	6.9	93.1	
Black	4.6	7.6	92.4	
Asian/Pacific Islander	15.1	9.4	90.6	
Latina	49.1	10.5	89.5	
Other race	3.9	14.0	86.0	
Education level				0.002
Less than high school	11.3	14.4	85.6	
High school diploma or GED	20.9	10.5	89.5	
Some college	33.2	9.9	90.1	
Bachelor’s degree or higher	34.7	6.5	93.5	
Insurance type				<0.001
Medi-Cal	47.9	12.9	87.1	
Private	45.0	5.8	94.2	
Other	7.1	8.3	91.7	
Age				0.042
18–24 years	21.4	11.0	89.0	
25–29 years	27.0	11.4	88.6	
30–34 years	29.3	8.1	91.9	
35 years and older	22.3	7.0	93.0	
Main language usually spoken at home				0.308
English	57.9	8.4	91.6	
Spanish	16.3	11.0	89.0	
English and Spanish equally	15.6	11.2	88.8	
Some other language	10.2	9.7	90.3	
Birth mode				0.014
Vaginal	69.6	10.6	89.4	
Planned cesarean	17.6	5.7	94.3	
Unplanned cesarean	12.8	7.8	92.2	
Gestational age				0.788
Preterm	6.5	11.6	88.4	
Early term	21.7	8.4	91.6	
Full term	57.1	9.3	90.7	
Late term	7.6	11.1	88.9	
Post term	7.1	9.0	91.0	
First baby				0.240
No	59.0	10.0	90.0	
Yes	41.0	8.5	91.5	
Midwife was main prenatal care provider				0.908
No	92.5	9.4	90.6	
Yes	7.5	9.1	90.9	
Obese prior to pregnancy				0.114
Not obese	72.4	8.9	1.1	
Obese	17.9	9.1	90.9	
BMI missing	9.7	13.5	86.5	

Results are weighted to be representative of singleton hospital births in California.

**Fig 1:**
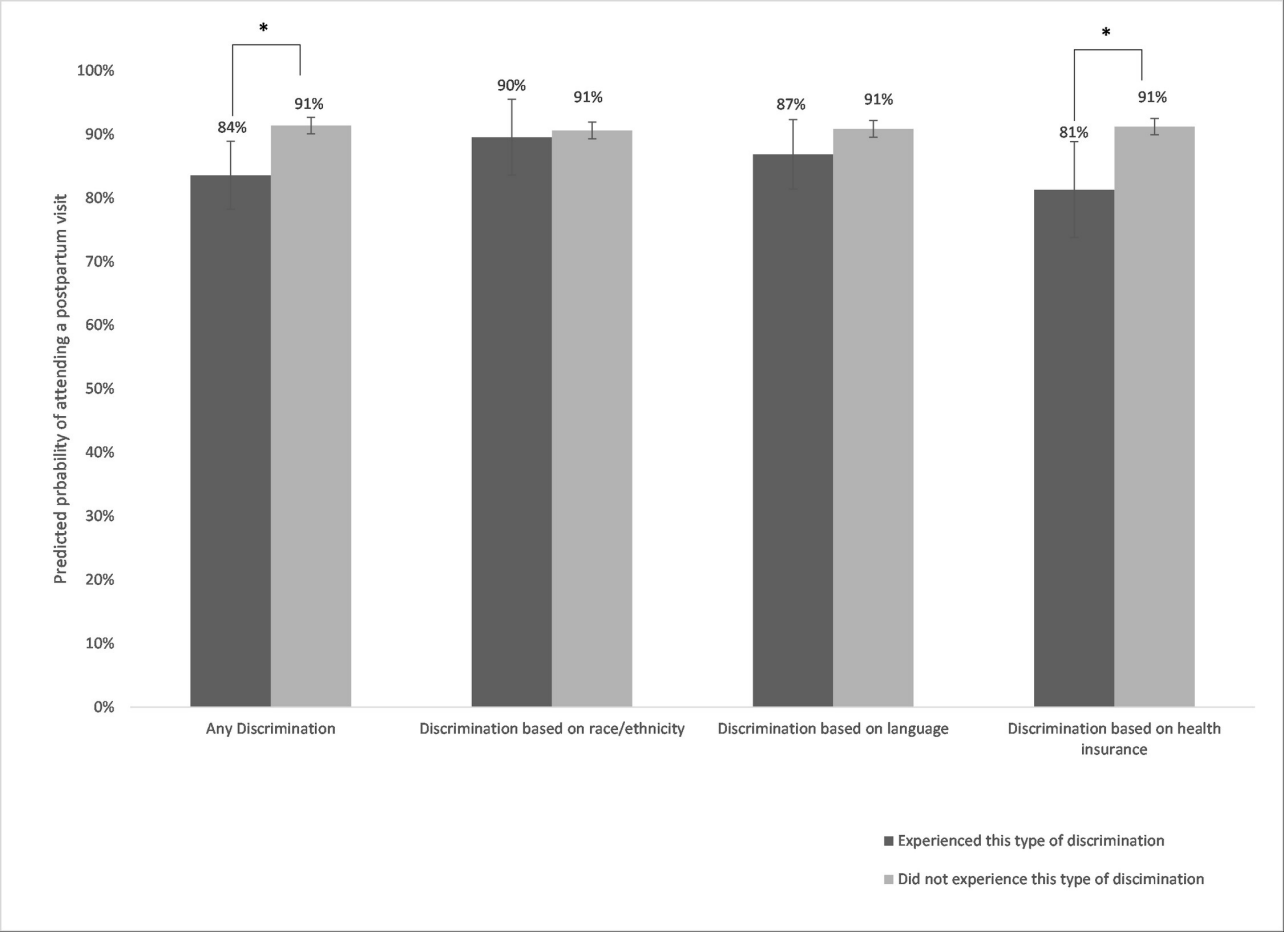
Predicted probability of postpartum visit by perceived discrimination. **Note**: *Indicates difference between the estimates is statistically significant with p<0.05. 95% confidence intervals are shown with vertical bars. Predicted probabilities are estimated from logistic regression models adjusted for race/ethnicity, insurance type, education, language, age, birth mode, gestational age, parity, prenatal care provider type, and pre-pregnancy obesity. Results are weighted to be representative of singleton hospital births in California.

Table 2 shows results for perceived discrimination and postpartum visit attendance. Nine percent of women reported experiencing one of the three types of perceived discrimination. In bivariate analyses, attending a postpartum visit was less likely for women who experienced discrimination based on language (84.6% vs. 91.0%, p = 0.02), discrimination based on health insurance (75.3% vs. 91.4%, p<0.001), and any discrimination (80.3% vs. 91.6%, p<0.001). Perceived discrimination based on race/ethnicity was not associated with postpartum visit attendance. In adjusted models, women who reported discrimination for any of the three reasons had a 7 percentage point (pp) lower predicted probability of attending a postpartum visit, compared to women who reported no discrimination (Fig 1). Women who experienced insurance-related perceived discrimination had a 10 pp lower predicted probability of postpartum visit attendance compared to women who did not experience this.

**Table 2:** Perceived discrimination during the childbirth hospitalization and postpartum visit attendance (N = 2,295).

	Total				
	Total (%)	Postpartum visit (%)	*P*	Adjustedǂ odds ratio (95% CI)	*P*
Any discrimination			<0.001		
No	91.4	91.6		1.00	
Yes	8.6	80.3		0.47 (0.30–0.73)	0.001
Discrimination based on race/ethnicity			0.349		
No	96.0	90.8		1.00	
Yes	4.0	87.7		0.88 (0.45–1.74)	0.720
Discrimination based on language			0.018		
No	94.0	91.0		1.00	
Yes	6.0	84.6		0.66 (0.39–1.11)	0.115
Discrimination based on health insurance			<0.001		
No	95.0	91.4		1.00	
Yes	5.0	75.3		0.41 (0.24–0.70)	0.001

ǂ Models controlled for race/ethnicity, insurance type, education, language, age, birth mode, gestational age, parity, prenatal care provider type, and pre-pregnancy obesity. Adjusted odds ratios are estimated from a separate logistic regression model for each discrimination type. Results are weighted to be representative of singleton hospital births in California.

**Fig 2:**
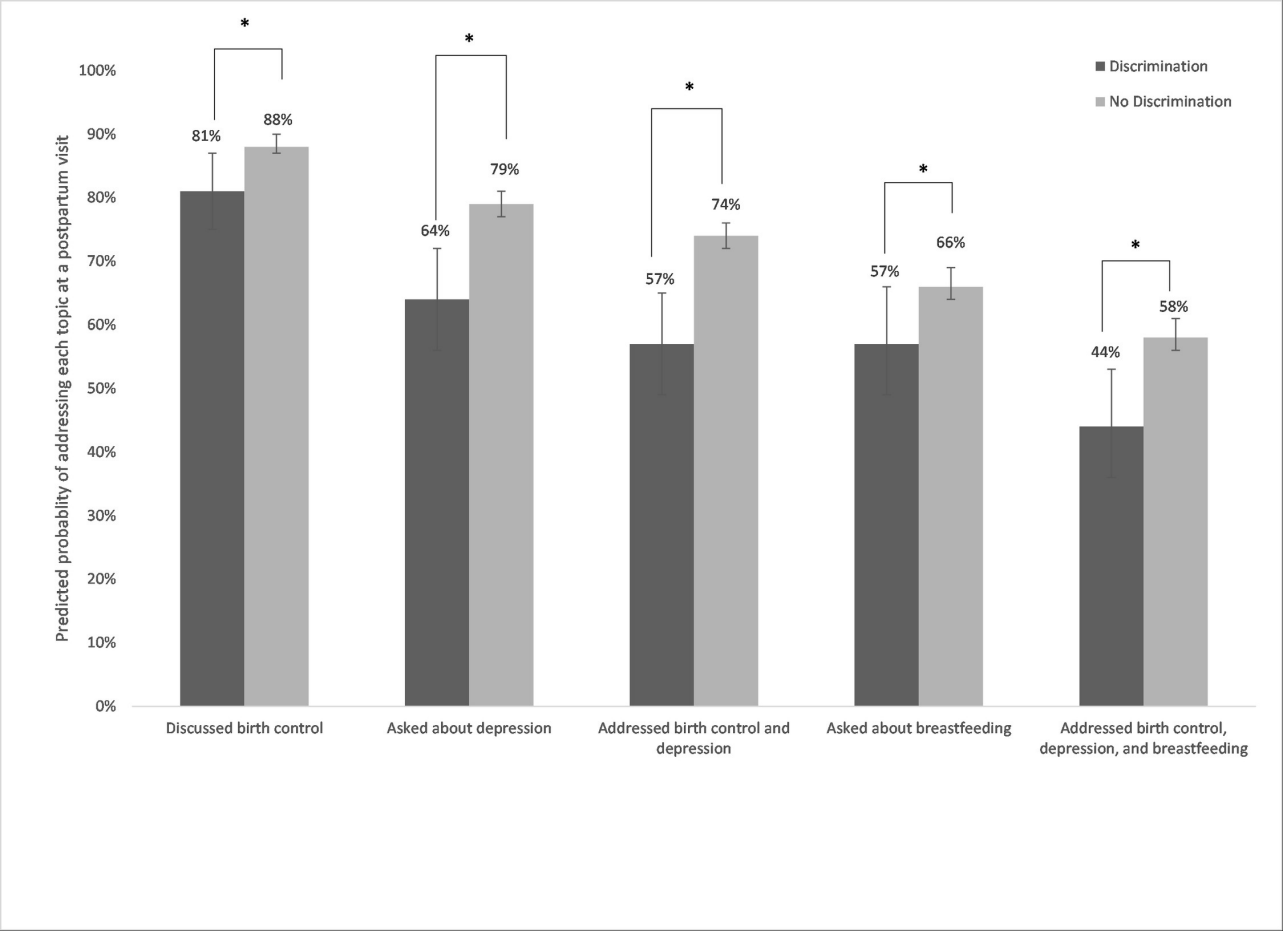
Predicted probabilities of topics addressed at postpartum visit by perceived discrimination. **Note**: *Indicates difference between the estimates is statistically significant with p<0.05. 95% confidence intervals are shown with vertical bars. Predicted probabilities are estimated from logistic regression models adjusted for race/ethnicity, insurance type, education, language, age, birth mode, gestational age, parity, prenatal care provider type, and pre-pregnancy obesity. Models including breastfeeding outcomes are limited to women who reported breastfeeding at 1 week postpartum. Results are weighted to be representative of singleton hospital births in California.

Table 3 reports topics addressed at postpartum visits by any perceived discrimination in the childbirth hospitalization. Overall, 87.6% of women who attended a postpartum visit reported that they discussed birth control with their provider. However, among women who reported discrimination, it was only 76.3% (p<0.001). This difference remained statistically significant in adjusted models. As shown in Fig 2, the predicted probability of discussing birth control during the postpartum visit was 81% for women who had experienced discrimination, versus 88% for women who had not. Overall, 78.4% of women reported that the clinician asked about depression at their postpartum visit. However, only 60% of women who reported discrimination during the birth hospitalization reported their clinician asking about depression. These differences persisted in adjusted models, with a 15 pp difference in the predicted probability of discussing depression by whether women reported discrimination (64% vs. 79%). Among women who were breastfeeding at 1 week postpartum, 65.9% reported that a clinician asked about breastfeeding at their postpartum visit. Women who experienced discrimination were less likely to report discussing breastfeeding at the postpartum visit (55.5%) compared to women who did not experience discrimination (66.8%). This association persisted in adjusted models, with a 9 pp difference in the predicted probability of discussing breastfeeding by perceived discrimination. Women who experienced discrimination were also less likely to report a provider discussing both birth control and depression at the postpartum visit, or birth control, depression, and breastfeeding (among those who were breastfeeding).

**Table 3:** Perceived discrimination during the childbirth hospitalization and topics addressed at the postpartum visit (N = 2,087).

		Experienced discrimination			Experienced discrimination	
	Total (%)	No (%)	Yes (%)	*P*	Adjusted^a^ odds ratio (95% CI)	*P*
Discussed birth control	87.6	88.5	76.3	<0.001	0.56 (0.36–0.88)	0.011
Clinician asked about depression	78.4	80.0	60.0	<0.001	0.45 (0.31–0.65)	<0.001
Addressed both birth control and depression	73.1	74.9	52.1	<0.001	0.44 (0.31–0.63)	<0.001
Clinician asked about breastfeeding^b^	65.9	66.8	55.5	0.009	0.67 (0.46–0.97)	0.035
Discussed birth control, depression, and breastfeeding^b^	57.7	59.0	41.4	<0.001	0.55 (0.38–0.80)	0.002

^a^Models controlled for race/ethnicity, insurance type, education, language, age, birth mode, gestational age, parity, prenatal care provider type, and pre-pregnancy obesity. Adjusted odds ratios are estimated from a separate logistic regression model for each discrimination type.

^b^Among those breastfeeding at 1 week postpartum, N = 1,917 Results are weighted to be representative of singleton hospital births in California.

The association of women’s characteristics with reasons for not attending a postpartum visit are shown in S1 Table. There were few statistically significant associations. Women experiencing perceived discrimination did not report different reasons for not attending the postpartum visit than those who did not experience discrimination. Younger women were more likely to report that they did not attend a visit for a reason other than not needing more care.

## Discussion

In this population-based sample of women who gave birth in California, over 90% attended a postpartum visit. However, women who experienced discrimination during the childbirth hospitalization were less likely to attend a postpartum visit, even after controlling for other characteristics. Importantly, our results also indicate that experiences of discrimination in the childbirth hospitalization are negatively associated with addressing recommended topics in the postpartum visit among women who do attend.

When we examined types of discrimination separately, we found that insurance-based discrimination was predictive of not attending a postpartum visit independent of insurance type. Recent studies have found that insurance-based discrimination is a relatively common form of perceived discrimination in healthcare settings, and in some cases is more common than race-based discrimination. Consistent with our findings, these studies have found that insurance-based discrimination is associated with delaying or not receiving needed care and receiving poor quality care [41–43].

We found that discrimination during the childbirth hospitalization not only made women less likely to attend a postpartum visit, confirming earlier findings from a national data source [19], but also less likely to discuss recommended topics during the postpartum visit with a clinician. All three topics examined in our study (contraception, depression and breastfeeding) were rated as very important and nearly always provided in a survey of clinicians who provide postpartum care [44]. Yet, a recent study examining documented postpartum visit content found that contraceptive counseling or provision occurred less than 50% of the time, while depression screening was documented in under 10% of visits, although provision of services did not vary by patient insurance status [27]. Our results suggest that experiences during the childbirth hospitalization may be another important factor predicting variation in postpartum visit content, and that further efforts to improve quality and equity in postpartum care are needed.

It is possible that the association between discrimination and postpartum visit content is due to differences in recall of the visit based on perceived discrimination. However, even if our findings are explained by differential recall rather than reflecting what took place during the clinical encounter, there could still be consequences for whether appropriate follow-up occurs. For example, if a woman experienced perceived discrimination and is thus less likely to remember a discussion with the clinician about contraception, she may also be less likely to fill a prescription or return for long-acting reversible contraception placement. Past experiences of discrimination in healthcare has been found to negatively impact communication quality in future encounters [28], and this could be a contributing factor in our findings as well.

### Limitations

There are several limitations to our study. First, the measure of postpartum visits used is based on having any office visits within 8 weeks postpartum, which is not perfectly analogous to a comprehensive postpartum visit. However, this definition is in line with other studies of postpartum care [10,11,19], and so is likely comparable to other survey-based studies of postpartum visit attendance. Second, the measure of postpartum visits is based on patient self-report, and it is possible that some respondents may have had a visit and not remember, or that there may have been social desirability bias leading to over-reporting of visits. Third, this data source did not contain information on medical conditions that may be relevant to postpartum care, such as diabetes, hypertension, and other pregnancy complications. However, we were able to include several relevant factors such as obesity status and birth mode in our adjusted results. Finally, while our multivariable models incorporated a range of characteristics, there may be other unmeasured differences between women who did and did not experience discrimination, which may also be associated with postpartum visit attendance. An important strength of this study is that survey respondents were a population-based sample of women who gave birth in California.

### Conclusions

As attention to the importance of postpartum care has increased, there is a need to better understand determinants of the likelihood of attending postpartum visits as well as care quality. Our findings highlight the ramifications that discrimination during the childbirth hospitalization may have into the postpartum period, leading not only to lower likelihood of receiving care but lower quality of postpartum care for those who attended visits.

## Supporting information

S1 Table Sample characteristics and reasons for not attending postpartum visit (n = 193). (DOCX)
