# Supplementary material for: Perceived discrimination during the childbirth hospitalization and postpartum visit attendance and content: Evidence from the Listening to Mothers in California survey
Source: PLoS One. 2021 Jun 23;16(6):e0253055. doi: 10.1371/journal.pone.0253055 (PMC8221509; doi:10.1371/journal.pone.0253055)
Supplement: S1 Table — (DOCX) [file pone.0253055.s001.docx]

| **Supplemental Table 1. Sample characteristics and reasons for not attending postpartum visit (n=193).** | | |
| --- | --- | --- |
|  | **Reason other than not needing more care** |  |
|  | **%** | ***P*** |
| Race/ethnicity |  | 0.935 |
| White | 59.6 |  |
| Black | 53.4 |  |
| Asian/Pacific Islander | 60.8 |  |
| Latina | 65.2 |  |
| Other race/ethnicity | 62.7 |  |
| Education level |  | 0.095 |
| Less than high school | 68.2 |  |
| High school diploma or GED | 77.7 |  |
| Some college | 55.3 |  |
| Bachelor's degree or higher | 56.0 |  |
| Insurance type |  | 0.128 |
| Medi-Cal | 68.4 |  |
| Private | 53.2 |  |
| Other | 49.8 |  |
| Age |  | 0.010 |
| 18-24 years | 78.2 |  |
| 25-29 years | 67.1 |  |
| 30-34 years | 44.8 |  |
| 35 years and older | 57.1 |  |
| Main langauge usually spoken at home |  | 0.462 |
| English | 59.3 |  |
| Spanish | 61.3 |  |
| English and Spanish equally | 75.1 |  |
| Some other language | 61.8 |  |
| Birth mode |  | 0.782 |
| Vaginal | 63.6 |  |
| Planned cesarean | 54.9 |  |
| Unplanned cesarean | 64.1 |  |
| Gestational age |  | 0.280 |
| Preterm | 86.2 |  |
| Early Term | 55.4 |  |
| Full Term | 61.3 |  |
| Late Term | 71.9 |  |
| Postterm | 55.6 |  |
| First baby |  | 0.524 |
| No | 64.6 |  |
| Yes | 59.8 |  |
| Midwife was main prenatal care provider |  | 0.775 |
| No | 63.1 |  |
| Yes | 59.3 |  |
| Obese prior to pregnancy |  | 0.523 |
| Not obese | 62.0 |  |
| Obese | 70.8 |  |
| BMI missing | 56.2 |  |
| Experienced any discimination during birth hospitalization |  | 0.553 |
| No | 63.8 |  |
| Yes | 58.1 |  |
| Experienced discrimination based on race/ethnicity |  | 0.354 |
| No | 63.7 |  |
| Yes | 49.4 |  |
| Ever experienced discrimination because of language |  | 0.835 |
| No | 63.1 |  |
| Yes | 60.5 |  |
| Experienced discrimination because of health insurance status |  | 0.991 |
| No | 62.8 |  |
| Yes | 63.0 |  |
| Results are weighted to be representative of singleton hospital births in California. | | |
